# Does Instruction Improve Older Adults’ Knowledge of Memory Aging?

**DOI:** 10.1093/geroni/igaa057.1168

**Published:** 2020-12-16

**Authors:** Rachael Turner, Celinda Reese, Susan Brigman, Katie E Cherry

**Affiliations:** 1 Oklahoma State University, Stillwater, Oklahoma, United States; 2 Franciscan Missionaries of Our Lady University, Baton Rouge, Louisiana, United States; 3 Louisiana State University, Baton Rouge, Louisiana, United States

## Abstract

Memory loss happens in later life. For cognitively healthy older adults, deficits in memory in everyday life may be frustrating, but are less severe compared to the memory dysfunction observed in persons with progressive dementia syndromes, such as Alzheimer’s disease (AD). Normal memory aging has been defined as benign memory deficits due to genuine maturational processes in otherwise healthy older adults. Pathological memory aging has been defined as memory dysfunction due to non-normative factors such as disease or trauma to the brain (Cherry & Smith, 1998). In the present research, we examined the effects of instruction on knowledge of memory aging issues among community-dwelling older adults. Participants ranged in age from 59 to 94 years. All were enrolled in a six-week lecture series on cognition in later life. They completed the Knowledge of Memory Aging Questionnaire (KMAQ: Cherry, Brigman, Hawley, & Reese, 2003) before and after the series. Results indicated that their knowledge of pathological memory aging was greater than their knowledge of normal memory aging, as expected. Importantly, both normal and pathological types of knowledge were impacted by instruction, as post-test scores were higher than pre-test scores for both scales. In addition, a select set of items reflecting ageist views were also impacted by instruction; scores on this subset were significantly improved (less reflective of ageist stereotypes) at the end of the lecture series. Implications for the design of educational programs on adult cognition for community-dwelling older adults are considered.

